# Energy-Efficient Integrated Electro-Optic Memristors

**DOI:** 10.1021/acs.nanolett.4c04567

**Published:** 2024-12-10

**Authors:** Yuhan He, Nikolaos Farmakidis, Samarth Aggarwal, Bowei Dong, June Sang Lee, Mengyun Wang, Yi Zhang, Francesca Parmigiani, Harish Bhaskaran

**Affiliations:** † Department of Materials, University of Oxford, Parks Road, Oxford OX1 3PH, U.K.; ‡ Institute of Microelectronics, Agency for Science, Technology and Research (A*STAR), 138634, Singapore; § Microsoft Research, 198 Cambridge Science Park, Cambridge CB4 0AB, U.K.

**Keywords:** electro-optic memristors, low energy in-memory computing, dual electrical−optical functionality, integrated photonics

## Abstract

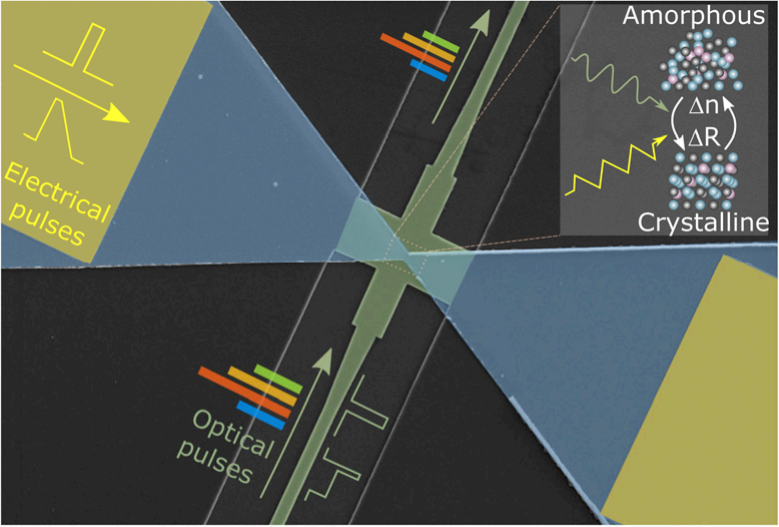

Neuromorphic photonic processors are redefining the boundaries of classical computing by enabling high-speed multidimensional information processing within the memory. Memristors, the backbone of neuromorphic processors, retain their state after programming without static power consumption. Among them, electro-optic memristors are of great interest, as they enable dual electrical–optical functionality that bridges the efficiency of electronics and the bandwidth of photonics. However, efficient, scalable, and CMOS-compatible implementations of electro-optic memristors are still lacking. Here, we devise electro-optic memristors by structuring the phase-change material as a nanoscale constriction, geometrically confining the electrically generated heat profile to overlap with the optical field, thus achieving programmability and readability in both the electrical and optical domains. We demonstrate sub-10 pJ electrical switching energy and a high electro-optical modulation efficiency of 0.15 nJ/dB. Our work opens up opportunities for high-performance and energy-efficient integrated electro-optic neuromorphic computing.

Neuromorphic computing, or brain-inspired computing, featuring co-located memory and processing units, is under extensive exploration for future high-throughput, energy-efficient computing systems.^1−3^ The key components, memristors,^4,5^ are a type of memory device that retain their state after programming without consuming additional energy, rendering them uniquely suited for low-power edge-computing applications such as in-memory sensing^6,7^ and multi-factor learning.^8,9^ Among them, electro-optic memristors^10^ are attracting attention because they enable programming and readout of the memory in both electrical and optical domains, i.e., providing dual electrical–optical functionality, bridging the efficiency of electronics and the bandwidth of photonics without requiring optical to electrical (OE) and electrical to optical (EO) conversions.

Phase-change materials, and notably the commonly employed alloy Ge_2_Sb_2_Te_5_ (GST), are exploited to modulate and store information in the optical transmissivity and electrical resistivity of different states (amorphous and crystalline), and have been widely used as both optical memristors^5,11−17^ and electrical memristors^18−21^ in computing systems, with tens to hundreds of picojoule optical programming energy and picojoule electrical programming energy. However, it has been challenging to implement phase-change electro-optic memristors in an integrated system because the mechanisms for optical and electrical programming are different. Evanescent coupling-based optical programming^11^ requires adequate interaction length, while threshold switching and Joule heating^18^ for electrical programming require small confined cells. The size mismatching between the electrical and optical domains poses hurdles in achieving both good heat confinement for electrical programming and sensing, and sufficient light–matter interaction for optical programming and sensing.

Two promising approaches have been explored to tackle this problem [Figure 1(a)]. The first is to enhance light–matter interactions at the nanometer scale by exploiting plasmonic structures. Such an approach was shown to provide excellent field confinement and demonstrate tens of picojoule-scale electrical switching energy.^22,23^ This is a highly promising approach but requires high resolution and a uniform fabrication and alignment process, posing challenges in scaling up. Alternatively, engineering the heat distribution in the electrical domain offers the possibility of aligning the size of programmable electronics with photonics. One such approach involves the use of external heaters combined with silicon waveguides to provide uniform Joule heating for electrically switching large-area phase-change materials.^3,24−27^ However, dual electrical–optical functionality has not been attained in these devices because their electrical readout is independent of the phase-change material state.

**Figure 1:**
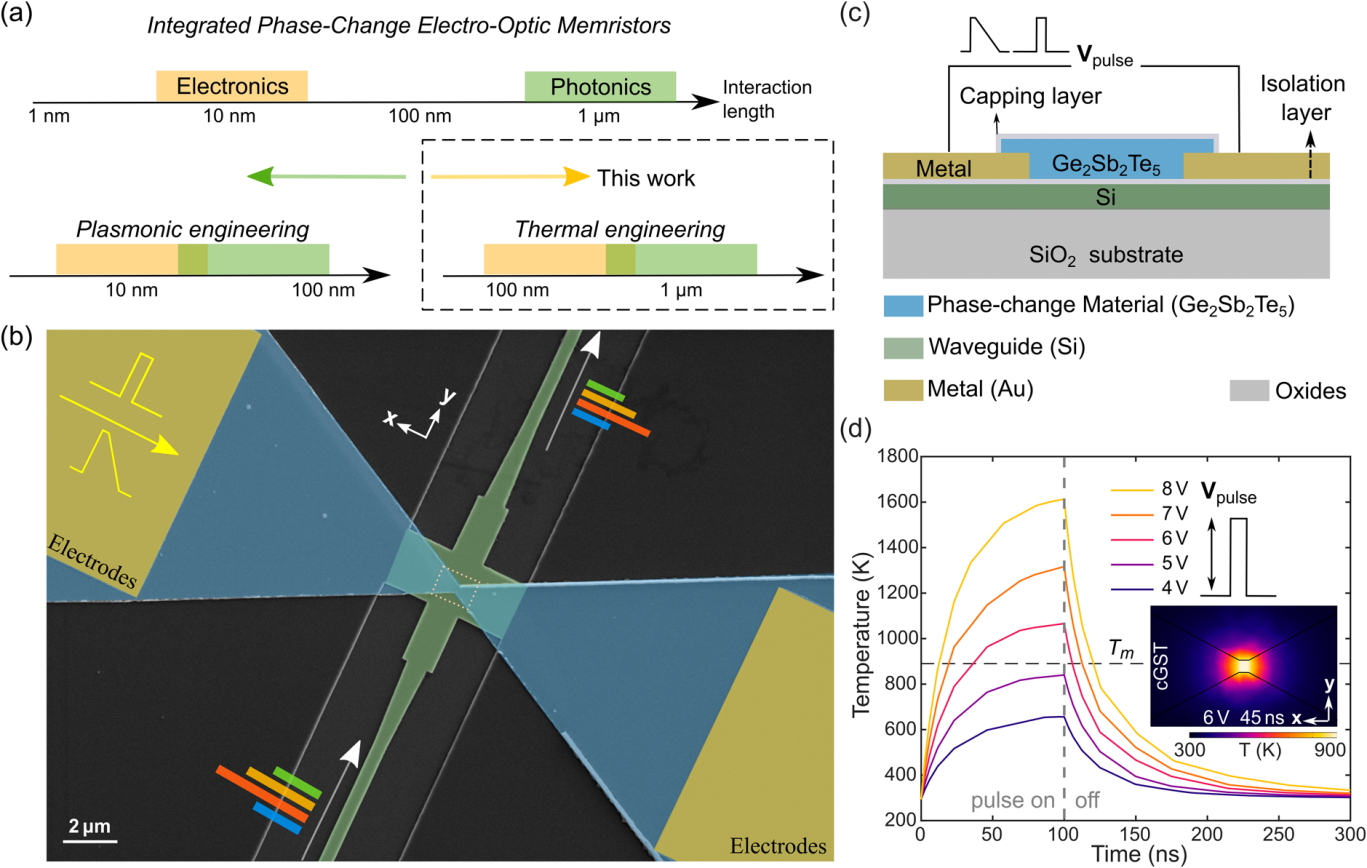
Schematic. (a) Concept of the thermal engineering design (this work) compared to the previous plasmonic engineering design. (b) False-color SEM image for a device with a 450 nm constriction (green: the waveguide with crossing structure; blue-gray: GST; dark-gold: gold pads). Scale bar: 2 μm. (c) Cross-section of the device center region. (d) Simulated temporal peak temperature curves for the GST region when applying square pulses on the pads with different voltage amplitudes and a fixed 100 ns pulse width. A pulse of 6 V with a 40 ns duration suffices to heat the GST above its melting temperature. *T*_*m*_: melting temperature for Ge_2_Sb_2_Te_5_ (∼890 K^29^). Inset: simulated temperature profile via COMSOL Multiphysics for the dashed square region in (b) after a 6 V, 45 ns amorphization square pulse. cGST: crystalline GST. Scale bar: 200 nm.

In the electronic domain, there has been huge progress in realizing low-energy electrical switching using thermal engineering techniques such as self-aligned carbon nanotube electrodes,^19^ superlattices,^20^ and self-confined cells.^28^ In this work, we apply these concepts to optoelectronics. We realize electrical heat confinement over a 100 × 100 nm region by tailoring the geometry of the phase-change material. Structuring the phase-change material as a nanoscale constriction, we demonstrate scalable and energy-efficient phase-change electro-optic memristors with dual electrical–optical functionality, paving the way for future fully integrated electro-optical in-memory computing systems.

Figure 1(b),(c) show a false-color SEM image and the cross-sectional schematic of our proposed device. The device combines a waveguide crossing structure with one-step lithography-defined phase-change material. The phase-change material (blue-gray) is designed as a bow-tie constriction structure with the narrowest feature size ranging from 100 to 450 nm. Electrical signals are supplied and read out via metal contact pads (dark-gold) away from waveguides, and optical signals via the waveguide underneath (green), coupled by an optimized crossing structure. Unlike plasmonic or doped-heater-based designs where the plasmonic structure or doping introduces intrinsic high loss,^3,22,23,25^ our crossing design induces less than 0.27 dB of insertion loss (Supplement 1 Figure S1).

When a voltage pulse is applied across the metal pads, the current-induced Joule heating will be confined to the narrow high-resistance region of the device, as validated through the FEM simulation profile in Figure 1(d). For a crystalline-state device, a voltage pulse of sufficient pulse amplitude and pulse width will melt-quench the phase-change material, leading to a phase transition to amorphous phase, i.e., switching of the device. The induced transmission and current change are read out simultaneously, and likewise the phase transition induced by optical stimuli (high-energy optical pulses) can also be read-out both electrically and optically. Details of the design parameters (thickness, constriction width, and constriction length) of the phase-change material geometry are presented in Supplement 1 Figure S2.

We further carried out heat and optical simulations to investigate the electrical switching performance and related optical response of our device. In Figure 2(a), the simulated light propagation profiles of the device with the GST in crystalline and amorphous states are presented, showing clear field contrast at the output ports (64% and 75%, respectively, normalized to the field at the input port). Here we focus on the amorphization process (simulated temperature profile for the crystalline pulse is presented in Supplement 1 Figure S3). With an increase in pulse amplitudes [Figure 1(d)] or widths [Figure 2(b)], a larger region of the material is heated up above the melting temperature, thus forming a larger amorphous region. Different volume ratios of amorphous and crystalline material provide intermediate electrical and optical readout levels between fully crystalline and fully amorphous states, enabling multilevel accessibility. To explore the relationship between device transmission and electrical switching energy, we built a model to calculate the broadband transmission response with different switched lengths [Figure 2(c)]. Here the switched length (*W*_*L*_) is defined as the length of the region with temperature over the melting temperature after an amorphization pulse. The transmission contrast (contrast normalization details in Supplement 1 Figure S4) increases with increased electrical switching energy, and a higher pulse amplitude provides lower minimum switching energy due to the shorter pulse width required [Figure 2(d)]. The simulated minimum switching energy for amorphization is less than 37 pJ (8 V, 20 ns).

**Figure 2:**
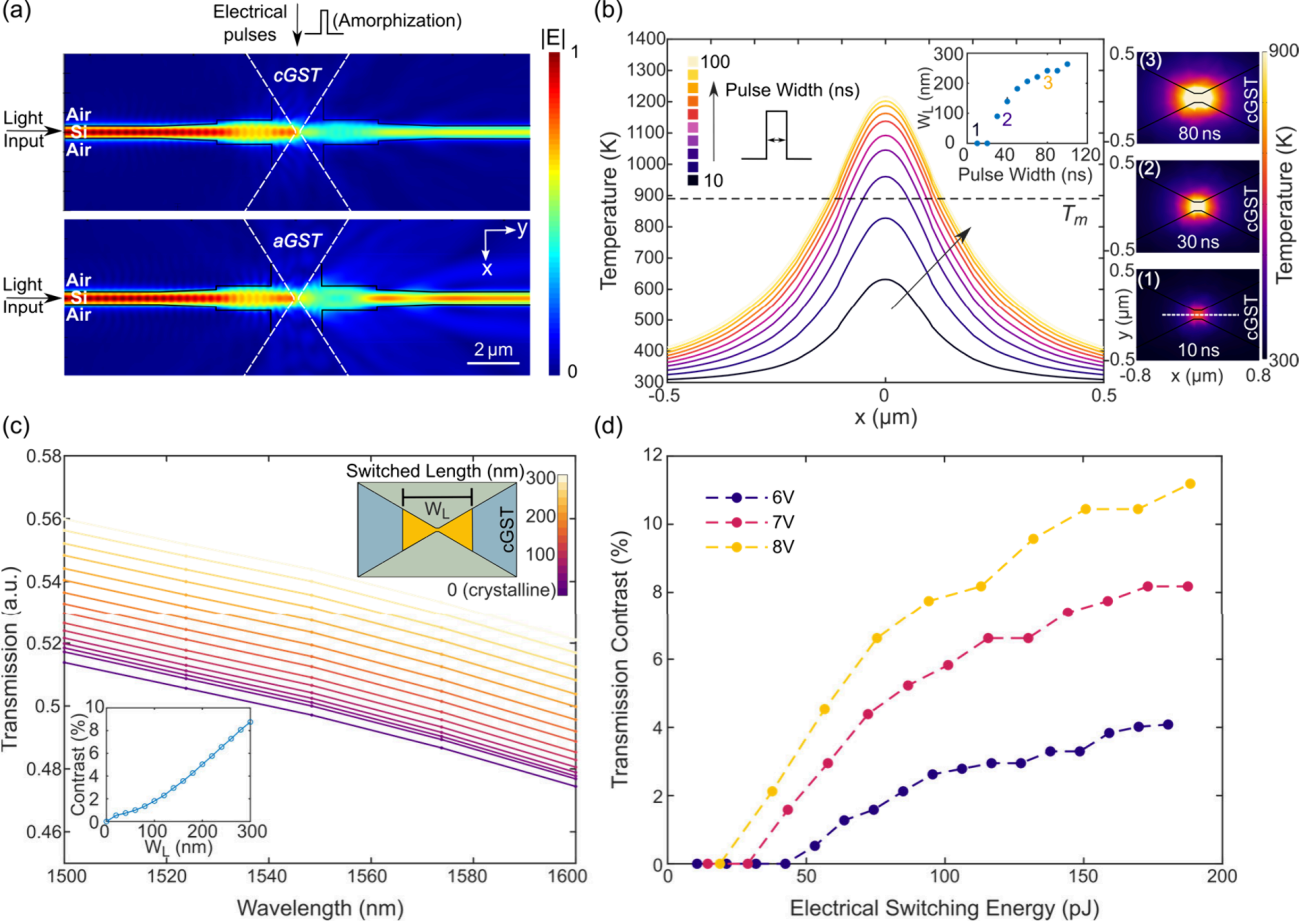
Simulated electrical switching performance. (a) Simulated (via Lumerical FDTD solutions) light propagation profiles of the devices with amorphous GST (aGST) and crystalline GST (cGST). The constriction width is 150 nm for the simulated device. (b) Simulated temperature distribution (via COMSOL Multiphysics) at a device *y*-cutline (white dashed line in inset (1)) when applying voltage pulses with different pulse widths (10–100 ns with a 10 ns increment, fixed amplitude at 7 V). Right column inset: corresponding simulated 2D temperature distribution for the device center slice with varied pulse widths (80, 30, and 10 ns, from top to bottom). Middle inset: relationship between voltage pulse width and device switched length (*W*_*L*_, device region with temperature over the melting temperature). The constriction width is 100 nm for the simulated device. (c) The simulated (via Lumerical FDTD Solutions) transmission change with different switched lengths. Inset: the relationship between switched length and transmission contrast at λ = 1574 nm. (d) Relationship between calculated electrical switching energy and transmission contrast based on (b) and (c). Pulse parameters are 6 V 10–170 ns, 7 V 10–130 ns, and 8 V 10–100 ns with a 10 ns increment.

After designing and fabricating the device (device characterization details in Supplement 1 Figure S5), we carry out experimental measurements for both electrical and optical switching based on a custom electro-optic setup. Devices are thermally annealed to the crystalline state before switching experiments.

We apply the electrical programming voltage pulse to the device’s metal pads. A low-power DC bias signal (100 mV unless otherwise specified) and probe light (λ = 1570 nm, 10 μW) are used to monitor the current and transmission, respectively (Methods and Supplement 1 Figure S6). In the binary switching measurements, we experimentally determine the device switching parameters for both amorphization and crystallization. We fix the amorphization pulse at 4.5 V and 30 ns (24 pJ including bias power) with a crystallization pulse as a 30 ns, 2.7 V pulse followed by a 250 ns triangular decay. Using these switching parameters, we demonstrate 25 cycles of sequential switching events with both the electrical and optical readout, as shown in Figure 3(a). The maximum current contrast is over 15%, and over 0.2% transmission contrast is obtained. The dynamic response of the electrical switching is presented in Supplement 1 Figure S7, where the device provides operational speeds of 60 and 290 ns (including post-excitation dead time^11^) for amorphization and crystallization, respectively. The re-set speed (amorphization) is faster than that of heater-based integrated phase-change electro-optical devices (>100 ns^3,24−27^), and the set speed (crystallization) is comparable (∼200 and 560 ns for P++ doped-Si heaters,^3,24^ >80 μs for PIN heaters,^25,26^ and >220 μs for graphene heater-based devices^27^). To show the reliability of our device, the cyclability test in Figure 3(b) demonstrates 100 cycles of reversible electrical switching with ∼20% current contrast. The confinement of heat to the constriction area results in low switching energy. Here the amorphization pulse (3.5 V, 10 ns) for electrical switching consumes only 2.1 pJ energy (switching current ∼58 μA).

**Figure 3:**
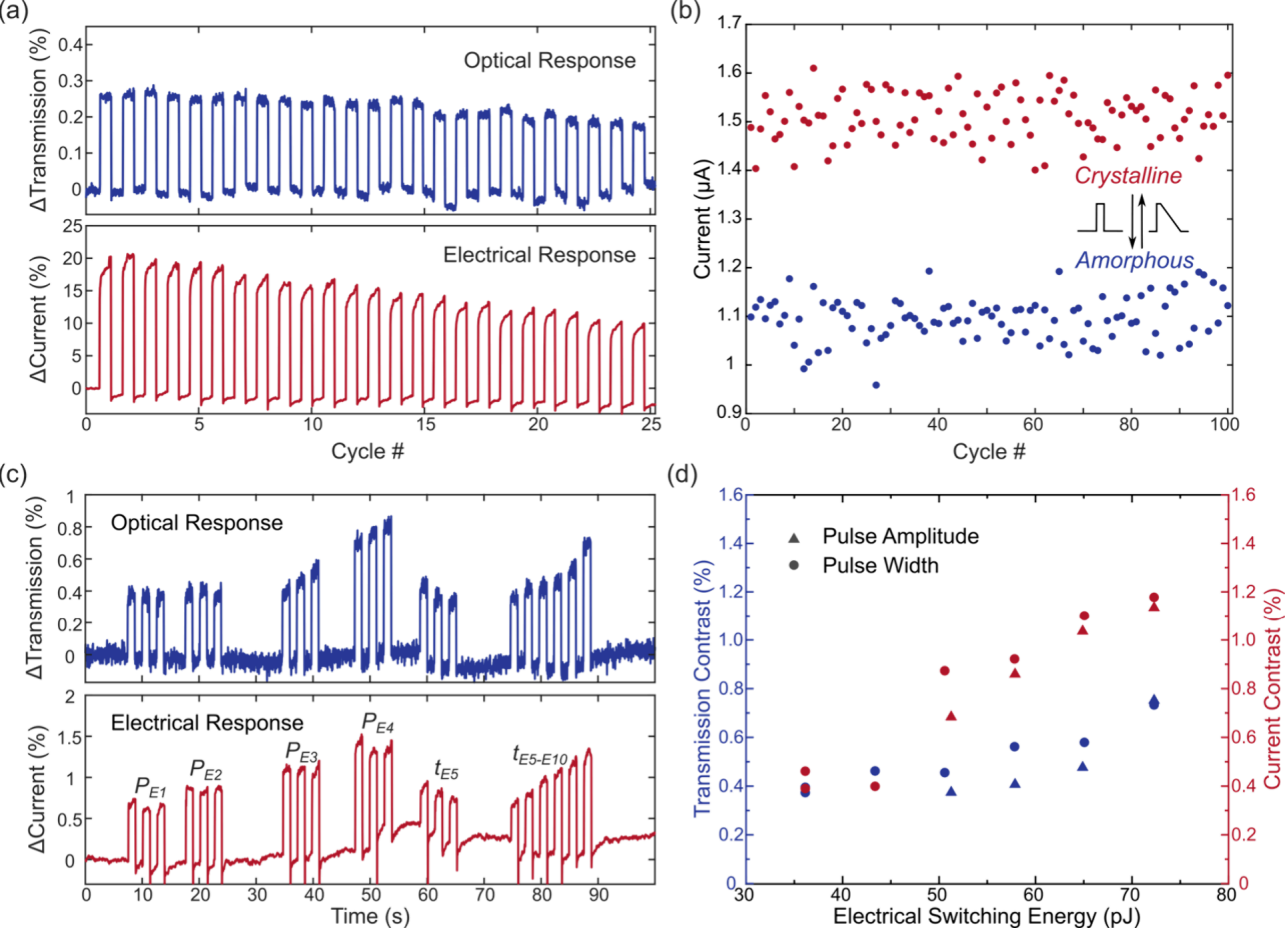
Electrical switching performance. (a) Experimental electrical switching with both optical and electrical readout for a device with a 120 nm constriction. The amorphization pulse is fixed at 4.5 V and 30 ns with a crystallization pulse as a 30 ns 2.7 V pulse followed by a 250 ns triangular decay. (b) 100 cycles of reversible electrical switching for a device with a 265 nm constriction. The amorphization pulse is fixed at 3.5 V with a 10 ns pulse width, and the crystalline pulse is a 2 V, 10 ns pulse with a 250 ns triangular decay tail. (c) Experimental multilevel electrical switching with both optical and electrical readouts for a device with a 130 nm constriction. *P*_*E1*_–*P*_*E4*_: amorphization pulse amplitude 8–9.5 V with a 0.5 V increment and fixed pulse width at 10 ns (each parameter repeated 3 times); *t*_*E5*_–*t*_*E10*_: 5–10 ns with a 1 ns increment and fixed pulse amplitude at 9.5 V. The crystallization pulse is fixed as an 8 V, 10 ns pulse followed by a 250 ns triangular decay tail. (d) Relationship between electrical switching energy and switching contrast for both transmission and current. The contrast is taken as the average of different cycles with the same amorphization pulse.

Next, to demonstrate multilevel switching, we fix the crystallization pulse as an 8 V, 10 ns pulse followed by a 250 ns triangular decay tail and vary both the amplitudes of amorphization pulses from 8 to 9.5 V (*P*_*E1*_–*P*_*E4*_ in Figure 3(c)) and the pulse widths from 5 to 10 ns (*t*_*E5*_–*t*_*E10*_ in Figure 3(c)). Distinguishable contrasts are obtained with increasing pulse widths or amplitudes, leading to higher contrast in both optical and electrical domains. The drift in the current levels/resistance can be attributed to the randomness of atomic rearrangement and structural relaxation of the amorphous phase.^30^Figure 3(d) illustrates the relationship between the increased contrast and increased pulse energy. The device switching energy obtained experimentally is similar to the simulated results in Figure 2(d) but with a lower transmission contrast. We attribute the deviation to the simplified simulation model, which only considers the switching region length, but the whole region is not fully switched in our experiments, leading to the smaller optical contrast.

More discussions about low-energy switching are presented in Supplement 1 Figure S8. We further demonstrate 50 cycles of electrical switching with 3% transmission contrast (5-fold improvement compared to our first plasmonic phase-change electro-optic memristor^22^) using a 19.5 pJ switching energy in Supplement 1 Figure S9. The electrically switched transmission contrast could be further enhanced with an optimized isolation layer or better coupling structure utilizing methods such as inverse design,^31^ which is beyond the scope of this work.

To demonstrate dual electrical–optical functionality, we further carried out optical switching measurements. We use amplified pump pulses (λ = 1571 nm) to program the device and the low-power probe light (λ = 1570 nm) to read out the device status. The current is monitored with the same DC bias signal as in electrical switching (Methods and Supplement 1 Figure S10). Switching pulse parameters are obtained experimentally. For binary switching, we fixed the optical pulse amplitude at 5.04 mW. A 25 ns pulse is used for amorphization, and a 10 ns, 1.51 mW pulse followed by a 250 ns rectangular tail for crystallization. We obtain successively reversible switching events for around 10% transmission contrast and over 40% current contrast [Figure 4(a)]. Cyclability test in Figure 4(b) demonstrates ∼3% reversible optical switching contrast with no obvious degradation after 100 cycles. We attribute the constant drift in the optical transmission to mechanical motion of the chip induced by the electrical probe assembly and to the formation of larger crystalline domains.^27^ After initial pulsing and amorphization of the phase-change material, ordered structures form which act as seeds for further crystal growth and are not fully amorphized with the same pulse.^32^ The optical transmission thus exhibits a trend toward a more crystalline state.

**Figure 4:**
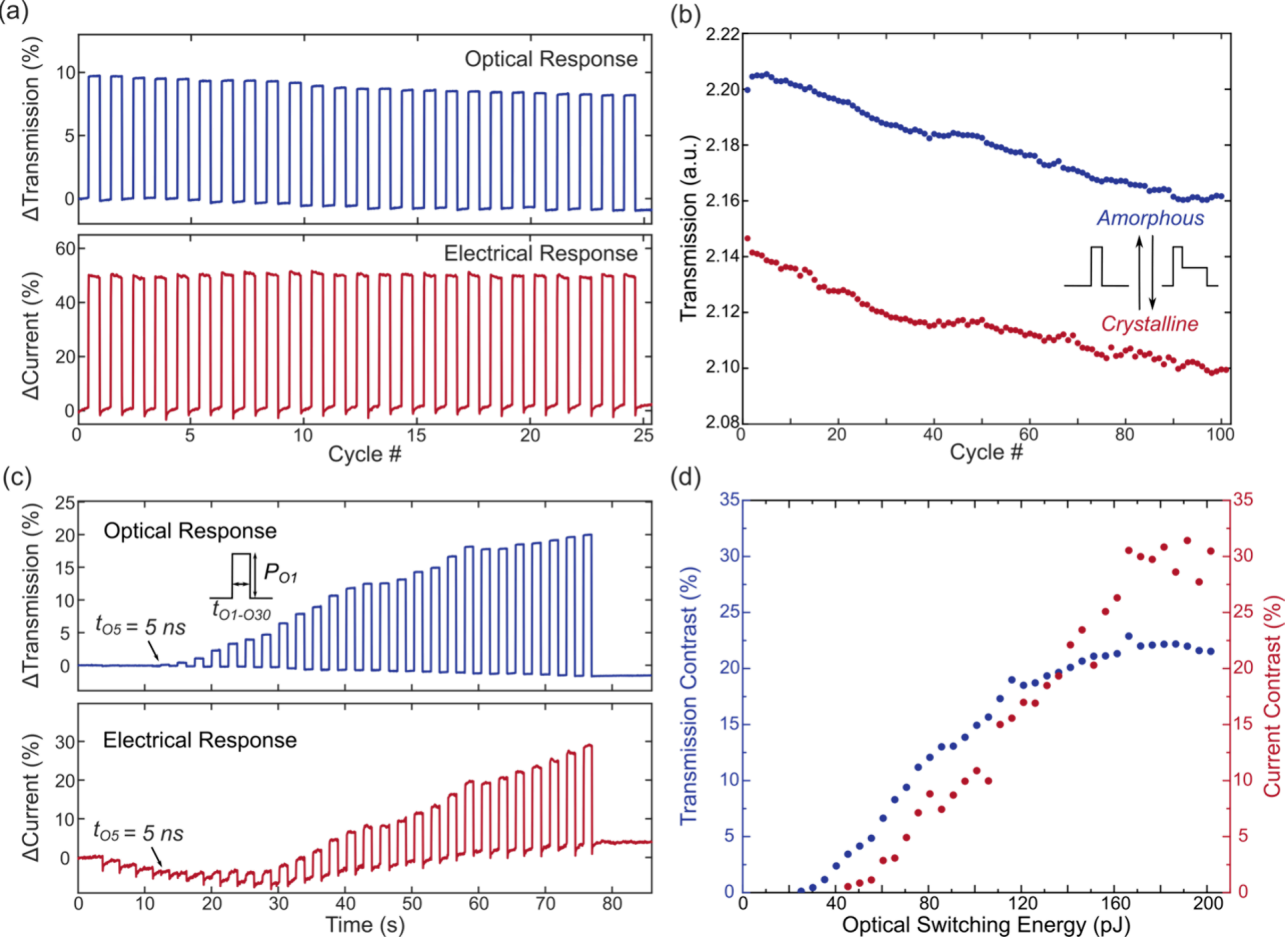
Optical switching performance. (a) Experimental optical switching with both optical and electrical readout for a device with a 310 nm constriction. The amorphization pulse is fixed at 5.04 mW with a 25 ns pulse width, and the crystalline pulse is a 5.04 mW, 10 ns pulse with a 1.51 mW, 250 ns rectangular decay tail. (b) 100 cycles of reversible optical switching for a device with a 110 nm constriction. The amorphization pulse is fixed at 14.39 mW with a 20 ns pulse width, and the crystalline pulse is a 14.39 mW, 5 ns pulse with a 6.48 mW, 250 ns rectangular decay tail. (c) Experimental multilevel optical switching with both optical and electrical readout for a device with a 310 nm constriction. Amorphization pulse amplitude is fixed at 5.04 mW (*P*_*O1*_) with pulse widths increasing from 1 to 30 ns (*t*_*O1*_–*t*_*O30*_) in 1 ns increments. The crystallization pulse is fixed as a 5.04 mW, 10 ns pulse followed by a 1.51 mW, 250 ns rectangular tail. (d) Relationship between optical switching energy and switching contrast for both transmission and current.

In addition to binary switching, multilevel switching is also achieved with optical programming. We fix the crystallization pulse as before and vary the amorphization pulse widths from 1 to 30 ns maintaining the amplitude at 5.04 mW [Figure 4(c)]. For short pulses (1–4 ns), the pulse power is not enough for amorphization, while the crystallization pulse further anneals the materials as indicated in the current trend. No optical transmission contrast is detected due to the limited signal-to-noise ratio. Increasing the amorphization pulse width to 5 ns induces over 0.05% transmission contrast using 25.2 pJ switching energy, and a 30 ns pulse achieves more than 20% contrast in both electrical and optical domains. Figure 4(d) further illustrates the relationship between the optical switching energy and switching contrast. The switching contrast increases first with increasing switching energy, then saturates at around 160 pJ with over 20% transmission contrast and around 30% current contrast. We attribute the much larger contrast for optical switching to the larger region switched by the optical pulses than by the electrical pulses.

Figure 5 provides a summary of the energy performance of our devices. The tens of picojoule switching energy we obtain for mixed-mode readouts, i.e., simultaneous readout in both electrical and optical domains) is similar to the plasmonic nanogap-based implementation and more than one order smaller than heater-based state-of-the-art integrated electro-optical phase-change devices. Table 1 highlights the significance of our work by comparing its metrics with those of other nonvolatile electro-optical devices based on GST. This work reduces the high insertion loss relative to plasmonic implementations (from ∼10 dB to 2–5 dB) and demonstrates very low electrical switching energy per unit modulation depth at 0.15 nJ/dB.

**Figure 5:**
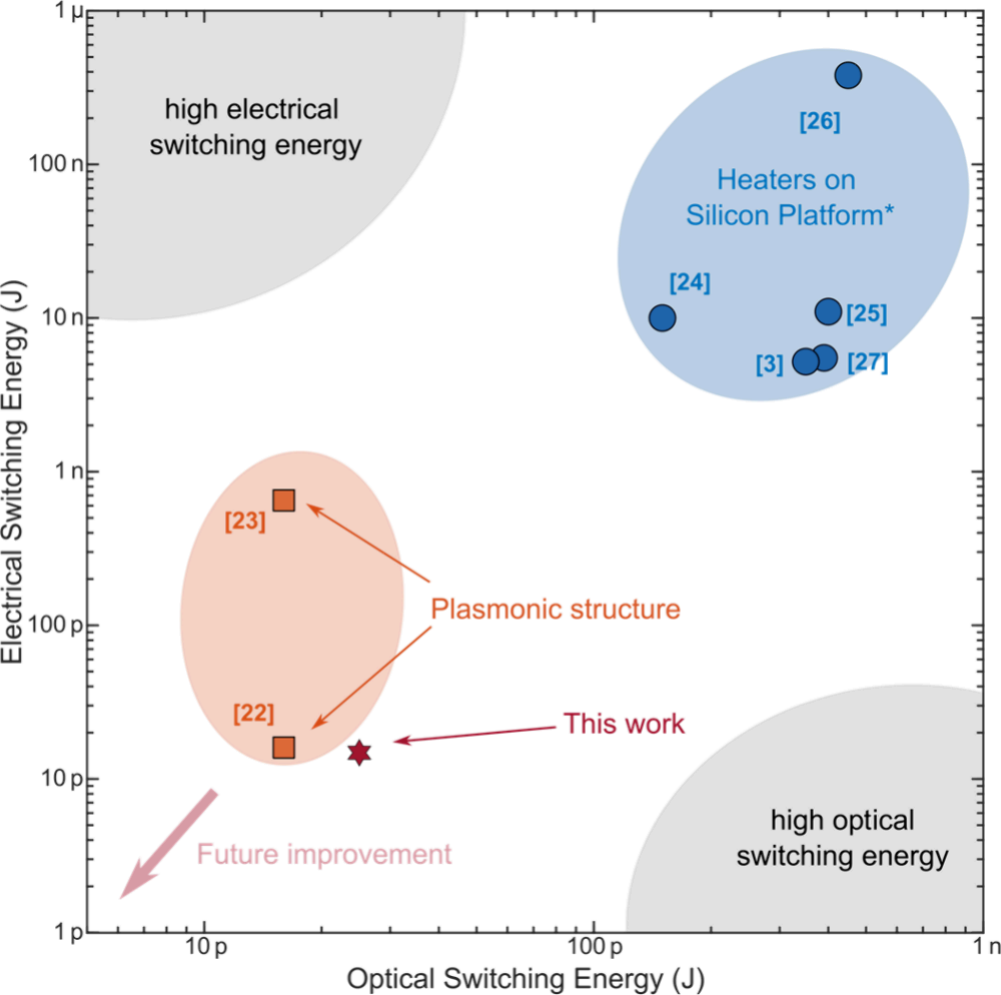
Switching energy map for different phase-change electro-optic memristor implementations. Heaters on a silicon platform: refs (3) and (24)–^27^. The optical switching energy is estimated based on ref (33). Plasmonic structures: based on refs (22) and (23).

**Table 1:**
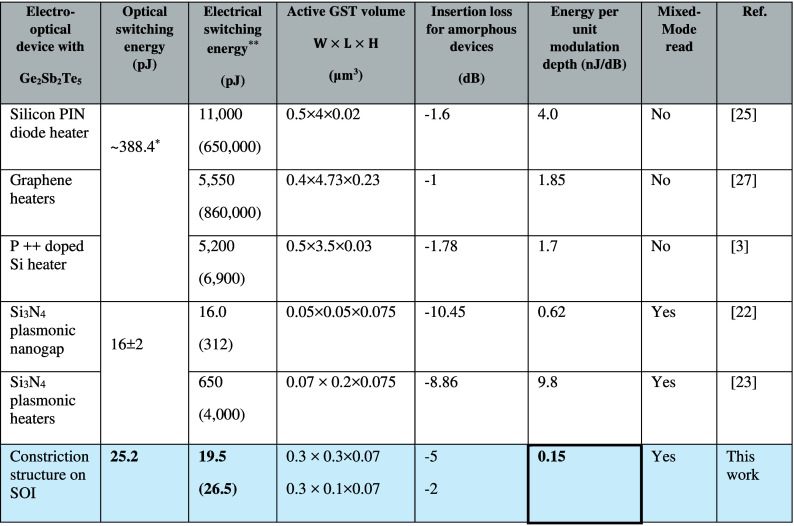
Performance Comparison of Electro-Optic Memristor Implementations Based on Ge_2_Sb_2_Te_5_^a^

a *Using the experimental data for 4 μm Ge_2_Sb_2_Te_5_ on Si from ref (33) as a reference. **Amorphization (crystallization).

In this work, we exploit the concept of thermal engineering to propose a novel design for implementing integrated phase-change electro-optic memristors. This is the first demonstration of integrated electro-optic memristors to achieve dual electro-optical functionality, low-energy switching, and potential for CMOS-compatible scaling at the same time. We confine the electrical heat profile of the device by carefully designing the phase-change material as a self-confined nanoscale constriction and combine the structure with photonics. This design achieves heat enhancement within the GST without placing constraints on the metallic layer, making the entire process foundry compatible and scalable for future applications. Benefiting from this thermal confinement, we enable electrical switching with tens to hundreds of μA programming current corresponding to ultralow electrical switching energy (sub-10 pJ), which is two orders lower compared with heater-based demonstrations. The devices show low energy for both electrical (19.5 pJ) and optical switching (25.2 pJ) with readouts in both electrical and optical domains, a strong modulation depth of 0.15 nJ/dB, and, importantly, multilevel operation. With further efforts to improve the device performance, this work will enable versatile electro-optical memory cells, which offer the potential for fully integrated, energy-efficient electro-optical computing systems combining in-memory programming and sensing abilities.

## Methods

### Sample Fabrication

The devices were fabricated on silicon-on-insulator (SOI) substrates with 220 nm Si on a 3 μm SiO_2_ layer. Waveguides, crossings, and grating couplers were patterned using electron-beam lithography (EBL, JEOL JBX5500 system) with a CSAR62 positive EBL resist, followed by reactive ion etching (RIE, Oxford Instruments) to achieve an etch depth of 110 nm with SF_6_ and CHF_3_ gases. Then 5 nm AlOx was deposited via atomic layer deposition (ALD, Savannah S200) for electrical isolation before EBL patterning and thermal evaporation of 5 nm Cr/75 nm Au for the metal pads. Finally, 150 nm of Ge_2_Sb_2_Te_5_ (GST) plus a 10 nm ZnS-SiO_2_ capping layer was patterned with a third EBL step and deposited with an RF sputtering system (PVD, AJA International Inc.). The devices were thermally annealed (250 °C for 5 min) to the crystalline state before switching tests.

### Measurement Setup

Optical switching experiments were performed with a pump–probe setup.^11^ A tunable laser (TSL-570, Santec) was employed to monitor the device transmission continuously (probe laser), and another laser source (N7711A, Keysight) was modulated with a function generator (AFG3102C, Tektronix) controlled electro-optical modulator (2623NA, Lucent Technologies) to send programming pulses (pump laser). The pump pulses were amplified via an erbium-doped fiber amplifier (AEDFA-CL-23, Amonics) before being coupled to the device. Circulators and filters (OTF-320, Santec) were used to separate the pump and probe signals and filter out the EDFA noise after amplification. All of the optical signals were collected via photoreceivers (model 2011 and model 2053, New Focus). The dynamic response of the device was amplified (AEDFA-PA-35-B-FA, Amonics) after a 99:1 splitter (TF1550R1A1, Thorlabs) and after the band-pass filter collected by the high-speed photoreceiver (model 1811, New Focus) and oscilloscope (TDS7404, Tektronix).

Electrical switching was realized using bias tee (ZFBT-4R2GW+, Mini-Circuits) to combine the RF signal from the above-mentioned pulse generator with the DC bias signal from a source meter (2614B, Keithley), and the pulses were sent to the device via RF probes (model 40A, GGB). The current was collected from a source meter.

## Data Availability

Data underlying the results presented in this paper are not publicly available at this time but may be obtained from the authors upon reasonable request.

## Supporting Information Available

Section S1. Design Parameters for the Waveguide Crossing; Section S2. Design Parameters for the Phase-change Material Constriction; Section S3. Simulated Temperature Profiles for Amorphization and Crystallization Pulses; Section S4. Normalization of the Transmission Contrast; Section S5. Device Characterization; Section S6. Measurement Setup for Electrical Switching; Section S7. Dynamic Optical Response of Electrical Switching; Section S8. Low-Energy Electrical Switching Performance; Section S9. Optical Readout of Electrical Switching; Section S10. Measurement Setup for Optical Switching (PDF)
