# Supplementary material for: Energy-Efficient Integrated Electro-Optic Memristors
Source: Nano Lett. 2024 Dec 10;24(51):16325–32. doi: 10.1021/acs.nanolett.4c04567 (PMC11674150; doi:10.1021/acs.nanolett.4c04567)
Supplement: Supplementary file 1 — nl4c04567_si_001.pdf [file nl4c04567_si_001.pdf]

Supplementary Materials for

Energy-efficient integrated electro-optic memristors

Yuhan He[1†], Nikolaos Farmakidis[1†], Samarth Aggarwal[1], Bowei Dong[1,2], June Sang Lee[1], Mengyun Wang[1], Yi Zhang[1], Francesca Parmigiani[3], and Harish Bhaskaran[*1]*

[1]Department of Materials, University of Oxford, Parks Road, Oxford OX1 3PH, UK.
[2]Institute of Microelectronics, Agency for Science, Technology and Research (A*STAR), 138634, Singapore
[3]Microsoft Research, 198 Cambridge Science Park, Cambridge CB4 0AB, UK.

*Corresponding authors: E-mail: *harish.bhaskaran@materials.ox.ac.uk*
[†]These authors contributed equally to this work.

S1. Design Parameters for the Waveguide Crossing

In the waveguide crossing design, we extend the MMI structure to the GST region to create planar devices, ensuring the GST film is deposited on the same height and maintaining good conductivity.

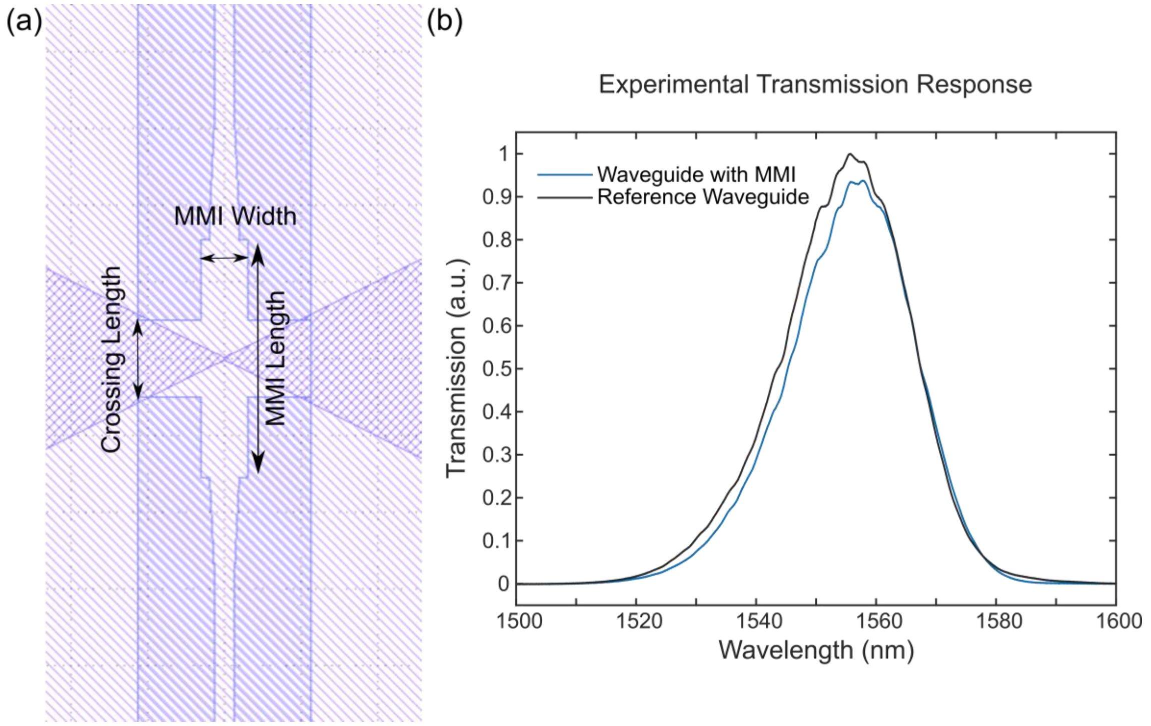

Fig. S1. Details for the multi-mode interferometer (MMI) crossing design**.** (a) Design parameters for the MMI structure. (b) Experimental transmission response for the optimized MMI design with crossing length = 2 μm, MMI length = 6.2 μm and MMI width = 1.2 μm. The bandwidth is limited by the grating couplers.

Based on this requirement, we fix the crossing length to 2 μm and optimize the other parameters of the MMI as indicated in Fig. S1(a) via Lumerical FDTD Solution to minimize the insertion loss. We obtain 94% (-0.27dB) experimental transmission for our device before phase-change material deposition, compared to 100% transmission for a plain waveguide on the same chip.

S2. Design Parameters for the Phase-change Material Constriction

The design parameters used in this work are 90 nm constriction length, 100-450 nm constriction width, and 150 nm thickness (Characterizations in Fig. S5(c)-(e)).

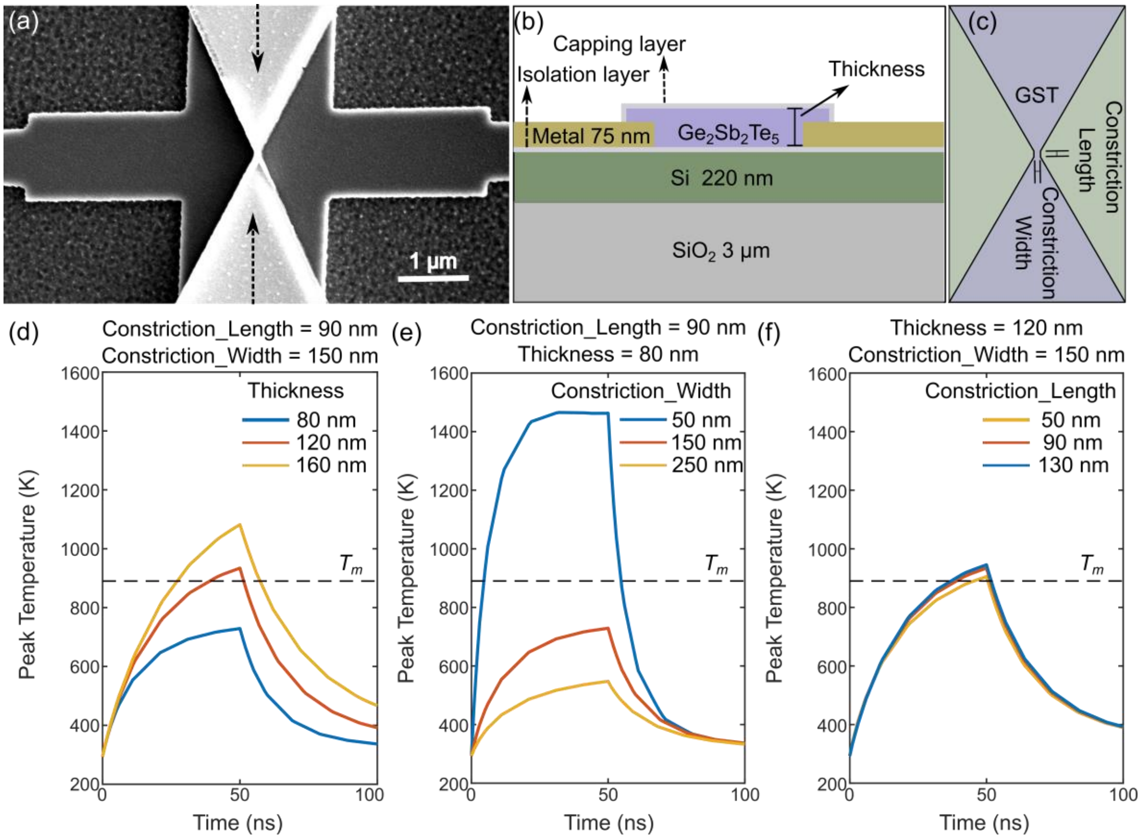

Fig. S2. Design parameters for the phase-change material constriction. (a) SEM image for the central region of the device. (b) Cross-section of the device at the dashed line in (a). (c) Top view of the device with indications of constriction width and constriction length. (d)-(f) COMSOL simulated temporal peak temperature curves for different material thickness, constriction width, and length with an 8-V, 50-ns pulse applied at the device metal contacts. For better comparison, we map the curve colors from higher device resistance (blue) to lower resistance (yellow).

We have conducted COMSOL simulations to explore how the above design parameters of the phase-change material geometry affect the electrical switching performance of the device. Fig. S2(d)-(f) plot the simulated temporal peak temperature profile of the phase-change materials region when an 8-V, 50-ns square pulse is applied to the metal electrodes.

Devices with higher thicknesses [Fig. S2(d)] exhibit lower resistance and provide larger current. Thus, the temperature rises faster to the melting temperature and provide faster switching speed. Conversely, devices with smaller constriction widths [Fig. S2(e)] exhibit higher resistance and lower current yet better heat confinement for the constriction region. The heat confinement accelerates the Joule-heating process for the constriction, enabling fast and low-energy electrical switching as well. Lastly, the effect of constriction length is not as significant as the other two parameters [Fig. S2(f)].

In summary, for fast and low-energy electrical switching, higher thickness and smaller constriction width is preferred. However, there is an upper thickness limit (around 150 nm) for the phase-change materials to achieve reversible switching, constrained by the required critical cooling rate [1]. Narrower constriction widths also limit the maximum transmission contrast (shorter evanescent-coupling length). Thus, the design of the constriction parameters must balance complex trade-offs among the parameters, with careful consideration of the specific application context. In the manuscript, we use 100-450 nm constriction width to balance the trade-off between switching energy and transmission contrast, and fix the constriction thickness at 150 nm, constriction length at 90 nm.

S3. Simulated Temperature Profiles for Amorphization and Crystallization Pulses

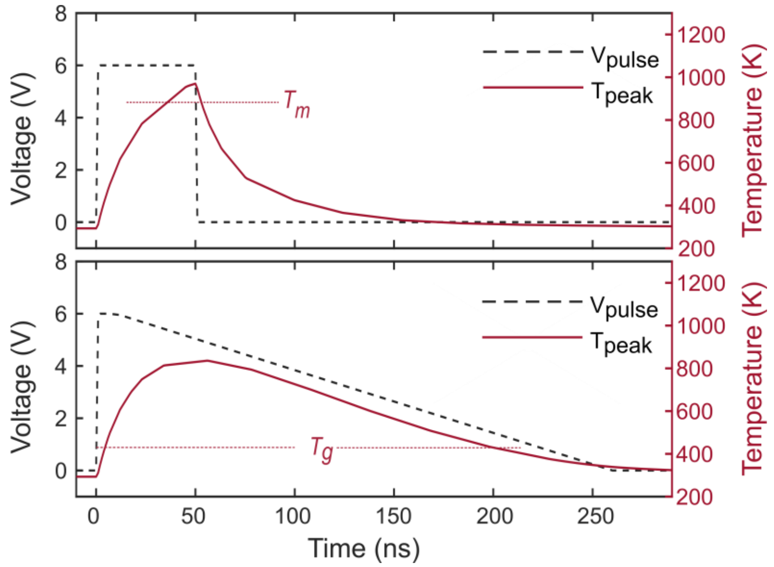

Fig. S3. Simulated (via COMSOL Multiphysics) temporal peak temperature profile during amorphization (6 V, 50 ns) and crystallization (a 6-V, 10-ns pulse followed by a 250-ns triangular decay) pulses for a device with a 100-nm constriction.

The short amorphization pulse rapidly heats the device above the melting temperature (T_m≈890 K [2]) of GST with a fast quenching, enabling the phase transition from crystalline to amorphous state. Similarly, the long triangular decay pulse heats up the device above the glass transition temperature (T_g≈415 K [2]) with a slow cooling-down, leading to crystallization.

S4. Normalization of the Transmission Contrast

In the main text, we define the transmission contrast as $\Delta T/T_{cry}$, where ΔT and T_{cry} represent the transmission change and the transmission for the crystalline state, respectively.

For smaller constriction widths (simulations in Fig. 2(c) and experiments in Fig. 3(a), Fig. 4(b)), switching from crystalline to amorphous increases the transmission, so we define $\Delta T= T_{amo} - T_{cry}$ (T_{amo} represents the transmission readout of the partially amorphous state). For larger constriction widths (experiments in Fig. 3(c)-(d), Fig. 4(a), Fig. 4(c)-(d), Fig. S7 and Fig. S9), pulse switching from crystalline to amorphous reduces the transmission, so we define $\Delta T= T_{cry} - T_{amo}$. We attribute the variation in the direction of the pulse-switched transmission change to the increased scattering

loss at the boundary between the amorphous and crystalline regions as the constriction width increases. The simulated transmission change for a representative device with a 300-nm constriction is shown in Fig. S4, where the transmission decreases with increased switched lengths, matching the experimental results in Fig. 4 and Fig. S9.

The current contrast is defined as $(I_{cry}\text{-}I_{amo})/I_{cry}$, where the crystalline state always providing higher current than the partially amorphous state.

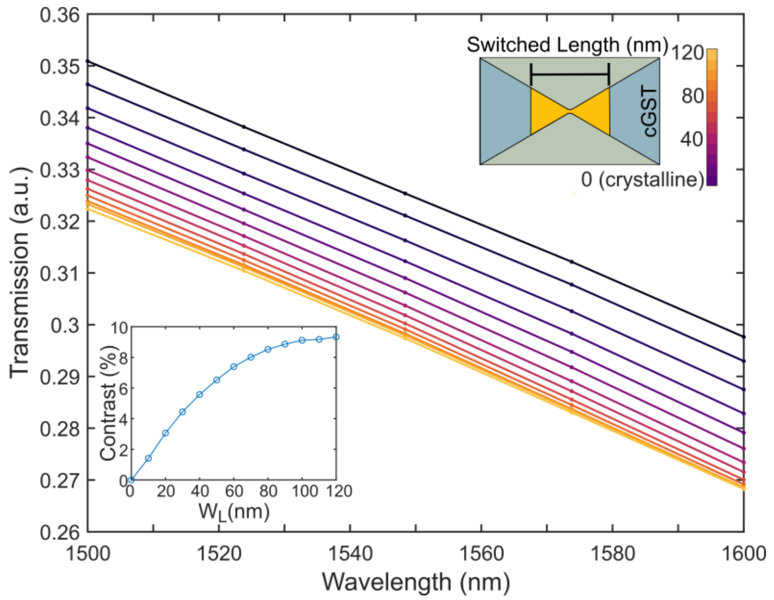

Fig. S4. The simulated (via Lumerical FDTD Solutions) transmission change with different switched lengths for a 300-nm constriction device. *Inset*: the relationship between switched length and transmission contrast at λ=1574 nm.

S5. Device Characterization

This section provides basic characterizations for the device. The loss of a typical device in the amorphous state (as-deposited) with a 100-nm constriction width is around 2 dB [Fig. S5(a)], increasing to 5 dB for a device with a 450-nm constriction width. Fig. S5(b) illustrates the current-voltage characteristics for a typical device in its two states, the device resistance dropping from 2.2 GΩ in its amorphous state to 11 kΩ in the crystalline state.

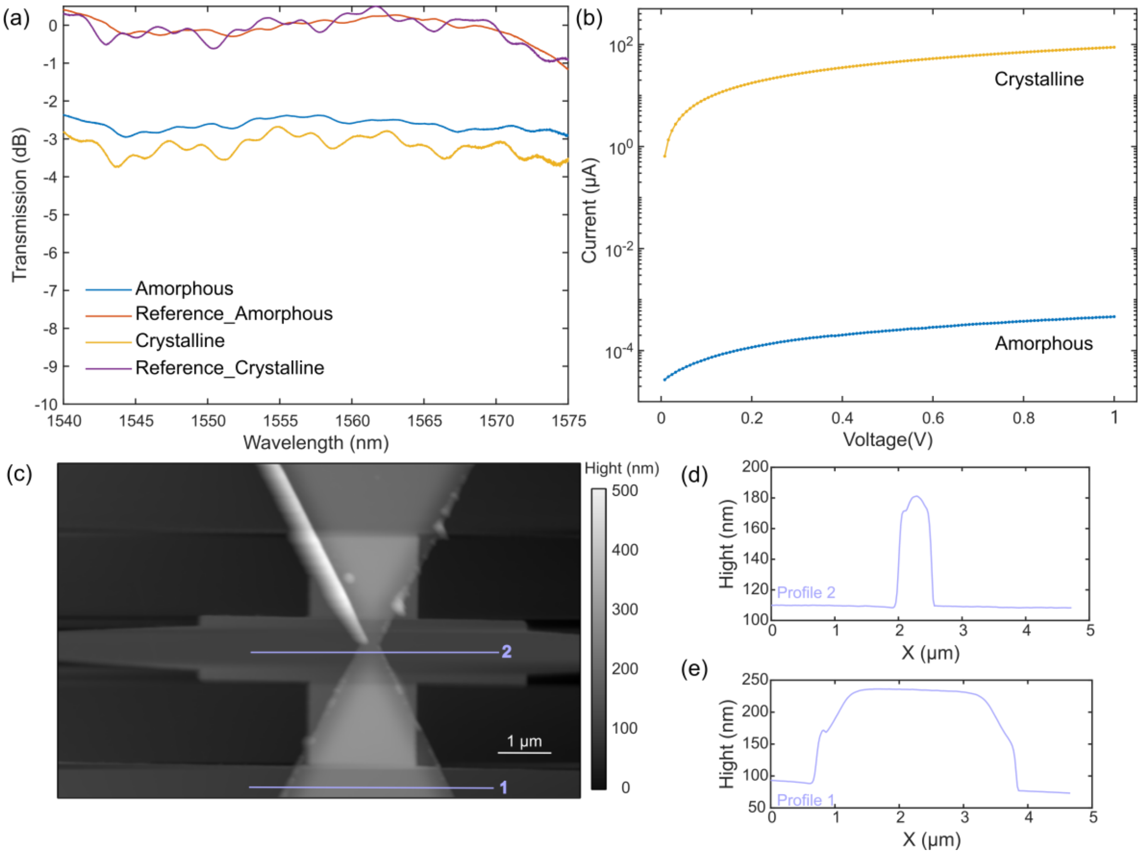

Fig. S5. Device characterization. (a) Transmission for a typical device in its two states with a 100-nm constriction width. Transmission is normalized to the transmission of plain waveguides with MMIs on the same chip. (b) IV measurements for a typical device in its two states with a 130-nm constriction width. (c)-(e) AFM image with thickness measurements.

We further collect atomic force microscope (AFM) micrographs [Fig. S5(c)] for the central region of a device. The height profiles in Fig. S5(d)-(e) demonstrate a height transition from around 150 nm (side, Fig. S5(e)) to 70 nm (center, Fig. S5(d)), from side to center, attributing to the shadowing effect during the RF sputtering process, which provides further heat confinement for

the central region, and explains the relative lower experimental switching energy compared with simulations.

S6. Measurement Setup for Electrical Switching

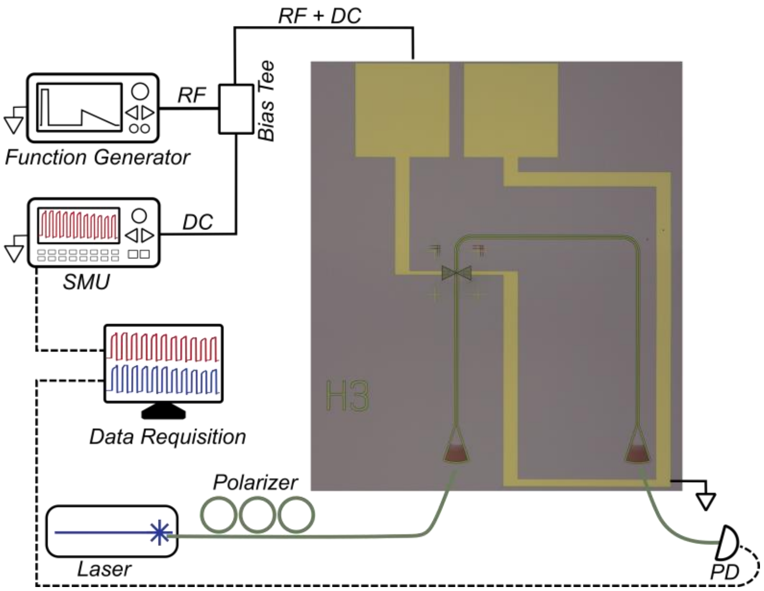

Fig. S6. Measurement setup for electrical switching. *SMU*: source measure unit. *PD:* photodetector.

S7. Dynamic Optical Response of Electrical Switching

Experimentally measured thermo-optic response of the constriction device is presented in Fig. S7. The post-excitation dead time [3] for a 30-ns amorphization pulse is calculated to be 30 ns, and the settling time for a crystallization pulse is 290 ns, providing operational speed of 60 ns and 290 ns for amorphization and crystallization, respectively.

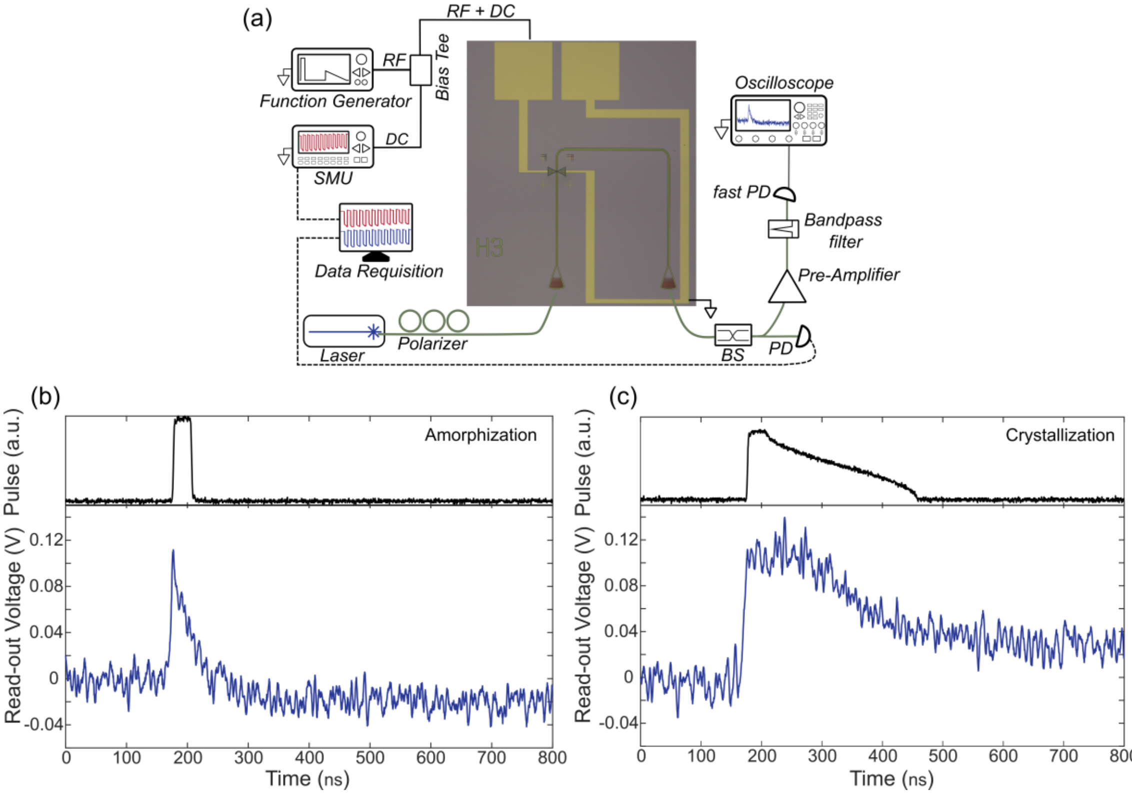

Fig. S7. Experimental dynamic response for the device with a 450-nm constriction. (a) Measurement setup. *SMU*: source measure unit; *PD:* photodetector; *BS*: 99:1 beam splitter, directing 99% of the output signal to the pre-amplifier and 1% to the PD. (b)-(c) Read-out voltage from the high-speed photodetector when applying a 9.5-V, 30-ns amorphization pulse and a 7.5-V crystallization pulse (a 30-ns square pulse followed by a 250-ns triangular tail). The read-out voltage curve is an average over 3 cycles.

S8. Low-Energy Electrical Switching Performance

Fig. S8(a) demonstrates over 100 cycles of stable reversible switching for 30% contrast. The amorphization switching energy is 10.4 pJ including DC bias power, providing higher switching contrast with a longer amorphization pulse (50 ns) compared to the results in Fig. 3(b) (10 ns for 20% contrast, 2.1 pJ). Fig. S8(b) provides multilevel switching results with increased amorphization pulse amplitudes. The amorphization energy increases from 224 fJ to 3.8 pJ, and the current contrast increases first and then saturates at around 70%.

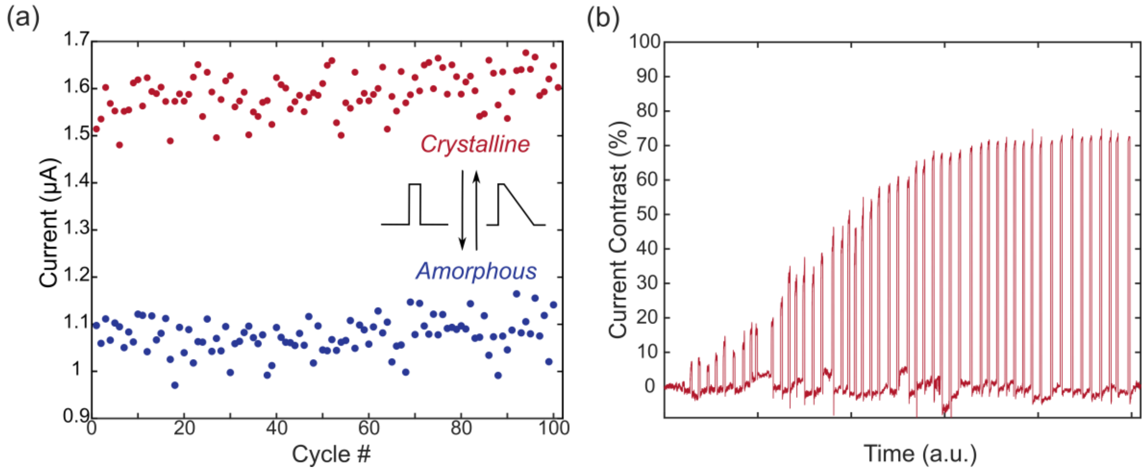

Fig. S8. Electrical switching performance. (a) 100 cycles of reversible switching for a device with a 265-nm constriction. The amorphization pulse is fixed at 3.5 V with a 50-ns pulse width, and the crystalline pulse is a 2-V, 50-ns pulse with a 250-ns triangular decay tail. (b) Multilevel electrical switching for a device with a 130-nm constriction. The amorphization pulse width is fixed at 25 ns with pulse amplitude varied from 1.5 V to 6.5 V (0.1 V increment at a time). The crystallization pulse width is a 25-ns pulse with a 250-ns triangular decay tail, with amplitude increasing from 1.5 V to 3.5 V. Device resistance is around 342 kΩ.

S9. Optical Readout of Electrical Switching

With 500-mV DC bias, Fig. S9(a) demonstrates 50 cycles of electrical switching with around 3% (0.13 dB) transmission contrast. The amorphization energy is only 19.5 pJ, which provides ultra-low energy consumption at 0.15 nJ/dB. Further multilevel switching results are presented in Fig. S9 (b), with the lowest amorphization energy (for the 7-V 30-ns pulse) calculated as 14.8 pJ. DC bias power is included in both energy calculations.

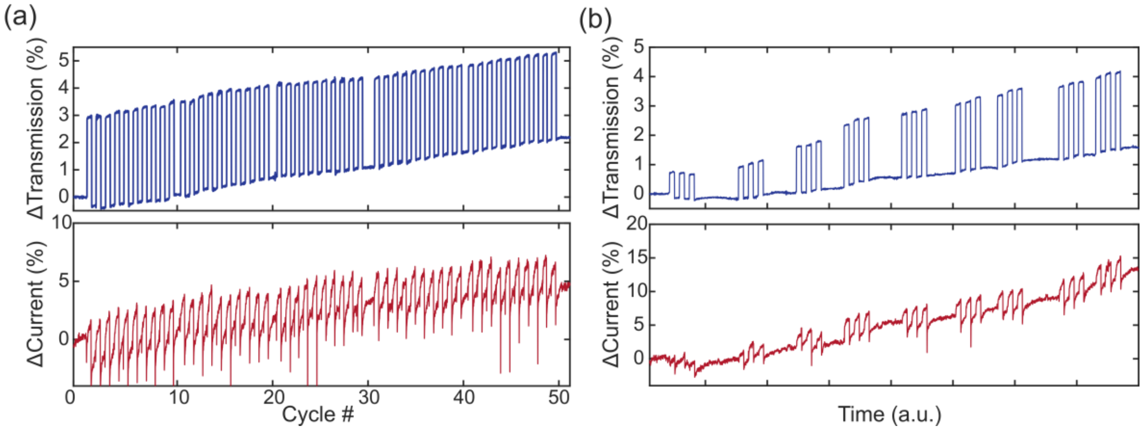

Fig. S9. Electrical switching with both optical and electrical readout for a device with a 310-nm constriction. (a) 50 cycles of reversible switching. The amorphization pulse is 9 V 30 ns and the crystallization pulse is fixed at 5 V, 30 ns with a 250-ns triangular tail, with 500-mV DC bias and 10-μW probe light power to readout current and transmission change, respectively. (b) Multilevel switching. Amorphization pulses are fixed at 30 ns with voltage amplitude varying as 7 V, 7.5 V, 8 V, 8.5 V, 8.6 V, 8.7 V, 8.8 V, 8.9 V, 9 V, each for three cycles. Crystallization pulses are the same shape as in (a) with amplitude increasing from 4.5 V to 5 V. The bias voltage and the probe power are the same as (a). Device resistance is around 143 kΩ.

S10. Measurement Setup for Optical Switching

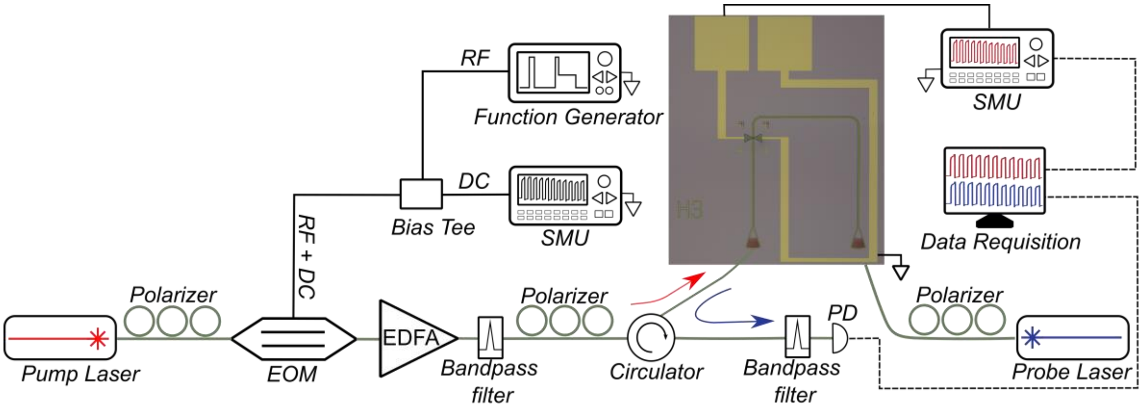

Fig. S10. Measurement setup for optical switching. *SMU*: source measure unit; *EOM*: electro-optical modulator; *EDFA*: erbium-doped fiber amplifier; *PD:* photodetector.

REFERENCES

1. Y. Zhang, C. Ríos, M. Y. Shalaginov, M. Li, A. Majumdar, T. Gu, and J. Hu, "Myths and truths about optical phase change materials: A perspective," Appl. Phys. Lett. **118**, (2021).

2. N. Yamada, E. Ohno, K. Nishiuchi, N. Akahira, and M. Takao, "Rapid-phase transitions of GeTe-Sb2Te3 pseudobinary amorphous thin films for an optical disk memory," J. Appl. Phys. **69**, 2849–2856 (1991).

3. C. Rios, M. Stegmaier, P. Hosseini, D. Wang, T. Scherer, C. D. Wright, H. Bhaskaran, and W. H. P. Pernice, "Integrated all-photonic non-volatile multi-level memory," Nat. Photonics **9**, 725–732 (2015).
